# Psychometric Validation of the Parental Stressor Scale: Neonatal Intensive Care Unit (PSS:NICU) in a Greek Cohort of Parents of Hospitalized Neonates

**DOI:** 10.3390/healthcare13212750

**Published:** 2025-10-30

**Authors:** Maria Tzeli, Maria Alexiou, Antigoni Sarantaki, Giannoula Kyrkou, Dimitrios Charalampopoulos, Sofia Biti, Marina Antoniadi, Aikaterini Fotiou, Anna Daskalaki, Tania Siahanidou, Christina Nanou, Dimitra Metallinou

**Affiliations:** 1 Department of Midwifery, School of Health and Care Sciences, University of West Attica, 12243 Athens, Greece; bombaki.ma@gmail.com (M.A.); esarantaki@uniwa.gr (A.S.); ikirkou@uniwa.gr (G.K.); dcharalampopoulos@uniwa.gr (D.C.); nanouxv@uniwa.gr (C.N.); dmetallinou@uniwa.gr (D.M.); 2 Neonatal Intensive Care Unit, IASO Maternity—Gynecology Clinic, 15123 Athens, Greece; mantoniadi@iaso.gr; 3 Department of Infectious Diseases, “Aghia Sofia” Children’s Hospital, 11527 Athens, Greece; 4 Nursing Directorate, IASO Maternity—Gynecology Clinic, 15123 Athens, Greece; nosil@iaso.gr; 5 Neonatal Intensive Care Unit, General and Maternity Hospital Helena Venizelou, 11521 Athens, Greece; katerinifg@hotmail.gr; 6 Third Department of Pediatrics, Attikon General Hospital, Medical School, National and Kapodistrian University of Athens, 12462 Athens, Greece; annadaskalaki3@gmail.com; 7 First Department of Pediatrics, “Aghia Sofia” Children’s Hospital, Medical School, National and Kapodistrian University of Athens, 11527 Athens, Greece; siahan@med.uoa.gr

**Keywords:** parental stress, stressor scale, neonatal intensive care unit, psychometric validation, cultural adaptation, translation, PSS:NICU, neonatal care, family-centered care, Greece

## Abstract

**Background/Objectives:** The Parental Stressor Scale: Neonatal Intensive Care Unit (PSS:NICU) is one of the most widely used tools for assessing parental stress in neonatal intensive care settings. This study aimed to translate, culturally adapt and validate the PSS:NICU in a Greek cohort. **Methods:** A multicenter, cross-sectional study was conducted with 150 parents (89 mothers, 61 fathers; mean age = 34.1 years, SD = 7.2) of hospitalized neonates from three Greek NICUs. The translation followed forward–backward procedures, expert review, and pilot testing. Data were analyzed for internal consistency, factorial validity, and group differences. **Results:** Confirmatory factor analysis supported the adequacy of the original three-factor structure (Sights and Sounds, Infant Behavior and Appearance, and Parental Role Alteration). Cronbach’s alpha and McDonald’s omega coefficients indicated excellent reliability for the total scale and its subscales. Female participants reported higher stress levels than males in most dimensions. **Conclusions:** The Greek version of the PSS:NICU demonstrated strong psychometric properties and cultural relevance. This adaptation provides a valid and reliable tool for assessing parental stress in Greek NICUs and facilitates cross-cultural comparisons and the development of targeted psychosocial interventions.

## 1. Introduction

Admission of a neonate to a Neonatal Intensive Care Unit (NICU) is a profoundly stressful experience for parents, who serve as the neonate’s primary attachment figures and emotional caregivers during this critical early developmental period [1]. The NICU environment—highly specialized, technologically advanced, and often perceived as intimidating—combined with parental fears regarding the neonate’s survival, potential complications, and long-term outcomes, substantially heightens stress levels among parents [2,3]. Parental distress during this period has been linked to heightened symptoms of anxiety, depression, and post-traumatic stress, as well as disruptions in bonding and later child socio-emotional outcomes [4,5,6]. Consequently, the accurate measurement of parental stress is a crucial component of family-centered neonatal care.

A variety of psychodiagnostic instruments have been developed to assess stress and related emotional states in parents of hospitalized infants. Generic measures such as the Perceived Stress Scale (PSS-10), Parenting Stress Index–Short Form (PSI-SF), State-Trait Anxiety Inventory (STAI), and Posttraumatic Stress Disorder Questionnaires, are frequently used in perinatal research [7,8,9]. While these tools demonstrate robust psychometric properties, they primarily capture generalized psychological distress rather than the unique environmental, sensory, and relational stressors of the NICU context. To address this gap, the Parental Stressor Scale: Neonatal Intensive Care Unit (PSS:NICU) was developed to specifically quantify the stress parents experience in response to the NICU environment, the neonate’s appearance and behavior, and alterations in parental role and caregiving identity [10].

Despite being developed over three decades ago, the PSS:NICU continues to be the most widely used and validated instrument for assessing NICU-related parental stress. Its structured domains align closely with contemporary conceptualizations of parental stress, and its cross-cultural adaptability has been demonstrated through multiple successful translations and validations in diverse linguistic and cultural contexts, including Turkish [11], Japanese [12], Italian [13], Portuguese [14], Swedish [15], Polish [16], Spanish [17], Arabic [18,19], and Persian [20]. It has also been adapted for use in the United Kingdom [21]. These studies consistently report strong internal consistency (Cronbach’s α = 0.56–0.98) and confirmatory evidence for its three-factor structure, underscoring the instrument’s enduring relevance and psychometric stability.

To date, no validated Greek version of the PSS:NICU exists, creating a gap in both research and clinical practice for assessing parental stress in Greek NICUs. Cultural adaptation and psychometric evaluation in this context are necessary to ensure conceptual equivalence, linguistic clarity, and measurement reliability. Addressing this gap will enhance the scale’s utility in Greek healthcare settings, enabling the development of culturally sensitive interventions to mitigate parental stress and promote family-centered care.

The aim of the present study was therefore to translate, culturally adapt, and psychometrically validate the PSS:NICU for use among Greek-speaking parents of neonates hospitalized in NICUs, and to examine its internal consistency and construct validity within this population.

## 2. Materials and Methods

### 2.1. Study Design

This was a multi-center, cross-sectional, methodological study aimed at translating, culturally adapting, and psychometrically validating the PSS:NICU for use in the Greek context. The study followed established guidelines for cross-cultural adaptation of self-report instruments, including forward–backward translation, expert panel review, pre-testing, and evaluation of psychometric properties [22,23]. Data were collected between May 2024–May 2025. The study design incorporated both exploratory and confirmatory factor analyses.

### 2.2. Participants

The study population comprised parents of neonates admitted to NICUs in three hospitals in Greece—one public, one university-affiliated and one private.

Inclusion criteria were the following: (a) age ≥ 18 years; (b) being a biological mother or father of a preterm neonate hospitalized in the NICU for a minimum of 5 consecutive days; (c) sufficient proficiency in reading and writing Greek to complete the questionnaire; (d) willingness to provide informed consent.

Exclusion criteria included the following: (a) parents of neonates with major congenital anomalies incompatible with life; (b) parents with a self-reported or documented severe psychiatric disorder that could impair questionnaire completion; (c) inability to participate due to medical or logistical reasons.

The sample size (n = 150) was considered adequate for confirmatory factor analysis, corresponding to approximately six participants per item. This ratio meets established psychometric recommendations of at least five participants per item for factor analytic procedures [24]. The final sample consisted of 150 parents (59.3% mothers, 40.7% fathers) with a mean age of 34.1 years (SD = 7.2). Most participants were between 31 and 40 years old (55.3%), married (91.3%), and of Greek nationality (99.3%). Nearly one-third were university graduates (31.3%), and half were employed in the private sector (50.7%). More than one-quarter (27.3%) reported having another child, while 5.3% had previous NICU experience. Obstetric data indicated that cesarean section was the predominant mode of delivery (91.3%), with preterm birth as the most common reason for NICU admission (82.7%). The mean gestational age at delivery was 32.1 weeks (SD = 3.3). As information on the exact number of previous pregnancies and births was not collected, parental reproductive experience was represented by the variable ‘having another child,’ which, given the timing of data collection during hospitalization, necessarily referred to children from prior pregnancies. Further sociodemographic and perinatal characteristics of the participants are summarized in Table 1.

### 2.3. Procedures

Participant recruitment took place during the neonate’s hospitalization, once the neonate was medically stable. Trained research assistants approached eligible parents in a private and quiet area within or adjacent to the NICU to ensure confidentiality and minimize external distractions.

Parents received verbal and written explanations of the study, emphasizing voluntary participation and the right to withdraw without affecting their infant’s care. Written informed consent was obtained from all participants before data collection commenced.

To ensure confidentiality, each participant was assigned a unique identification code. Completed questionnaires were stored in locked cabinets accessible only to the research team, and electronic data were entered into a secure, password-protected database. No identifying information was recorded in any reports, presentations, or publications derived from the study.

Participants first completed a brief sociodemographic questionnaire, including gender, age, family status, education level, nationality, employment, existence of other children, and previous experience in NICU setting, as well as a clinical information questionnaire, including weeks of gestation at birth, mode of delivery, gender of the neonate, birth weight and Apgar score. Subsequently, they completed the Greek-translated version of the PSS:NICU.

To evaluate content validity, the translated PSS:NICU was reviewed by a panel of three experts: a neonatologist and head of a NICU, an experienced NICU midwife, and a psychologist. The experts evaluated each item of the questionnaire for its relevance and comprehensiveness, and changes were made in line with their recommendations. All experts confirmed that the final items were conceptually relevant and culturally appropriate for use in the Greek context. The prefinal version was pilot-tested in a convenience sample of 10 parents, who confirmed item clarity and comprehensibility, leading only to minor wording adjustments and no major modifications [15,25].

### 2.4. Research Instrument

The Parental Stressor Scale: Neonatal Intensive Care Unit (PSS:NICU) is a 26-item self-report instrument developed to assess the intensity of stress experienced by parents during their neonate’s hospitalization in the NICU [10]. The scale measures stress across three conceptual domains:

1. Sights and Sounds (5 items)—evaluates stress related to the sensory and technological environment of the NICU (e.g., equipment alarms, lighting, medical devices).
2. Infant Behavior and Appearance (14 items)—captures stress associated with the neonate’s physical appearance, medical interventions, and observable behaviors.
3. Parental Role Alteration (7 items)—reflects stress due to disruption of the expected parenting role, including restrictions in providing care or physical contact.

Items are rated on a 5-point Likert scale ranging from 1 (“not at all stressful”) to 5 (“extremely stressful”), with higher scores indicating greater perceived stress. The original English version of the PSS:NICU has demonstrated satisfactory psychometric properties, including good internal consistency (Cronbach’s α values typically ranging from 0.80 to 0.94) and evidence of construct validity across diverse populations.

Permission for the translation and use of the instrument in this study was obtained from its original author, Dr. Miles.

### 2.5. Ethics

The study protocol was reviewed and approved by the Research Committees of the participating hospitals and by the Research Ethics Committee of University of West Attica, given the active involvement of its academic staff and postgraduate students (PhD and MSc candidates) in the research team.

All procedures complied with the ethical principles outlined in the Declaration of Helsinki and its later amendments, as well as with applicable national regulations and the European Union’s General Data Protection Regulation (GDPR).

### 2.6. Data Analysis

Quantitative variables were summarized as mean values with standard deviations (SD) and as medians with interquartile ranges (IQR), while categorical variables were presented as absolute and relative frequencies. Sampling adequacy was assessed using the Kaiser–Meyer–Olkin (KMO) measure. Factor loadings of ≥0.40 and Eigenvalues ≥ 1.00 were considered the minimum thresholds for item retention.

A Confirmatory Factor Analysis (CFA) using the maximum likelihood method was performed to examine how well the hypothesized three-factor structure fits the data. During parameter estimation, the variance of the latent variables was fixed at one, and the factors were allowed to correlate. Model fit was evaluated using several indices: chi-square to degrees of freedom ratio (χ^2^/df), comparative fit index (CFI), Tucker–Lewis index (TLI), standardized root mean square residual (SRMR), and root mean square error of approximation (RMSEA) [26]. For the CFI and TLI, values close to or above 0.90 indicate acceptable fit, while values ≥ 0.95 suggest excellent fit. The CFI is generally preferred because it accounts for sample size. RMSEA values < 0.05 indicate good fit, and values up to 0.08 reflect acceptable fit. SRMR values < 0.08 and χ^2^/df ratios below 3 were also considered indicators of good model fit [27,28].

Internal consistency reliability was assessed using Cronbach’s alpha and omega coefficients. Values ≥ 0.70 were considered acceptable for research purposes [29]. Convergent validity was evaluated by examining intercorrelations (Pearson’s r) among subscales of the PSS:NICU belonging to the same questionnaire.

Independent-samples *t*-tests were performed to evaluate the association between scores and reasons for NICU admission, and to determine differences between male and female participants.

Data completeness was very high. At most, two missing values were observed for a few items of the scale. In accordance with the scoring guidelines of the PSS:NICU [10], subscale scores were calculated based on the available items, and no imputation was required [18,21].

All *p*-values were two-tailed, with statistical significance set at *p* < 0.05. Data analyses were conducted using SPSS statistical software (version 27.0; IBM Corp., Armonk, NY, USA and STATA (version 15.0; StataCorp, College Station, TX, USA).

## 3. Results

Mean responses to the PSS:NICU items, together with their corrected item–total correlations and reliability coefficients (Cronbach’s α if item deleted) are presented in Table 2. The highest stress levels were reported for “being separated from their neonate”, followed by “not being able to hold their neonate when desired” and “feeling helpless and unable to protect their neonate from pain or painful procedures”. All corrected item–total correlations exceeded 0.30, confirming that each item contributed adequately to its corresponding subscale and that no item required removal.

**Figure 1:**
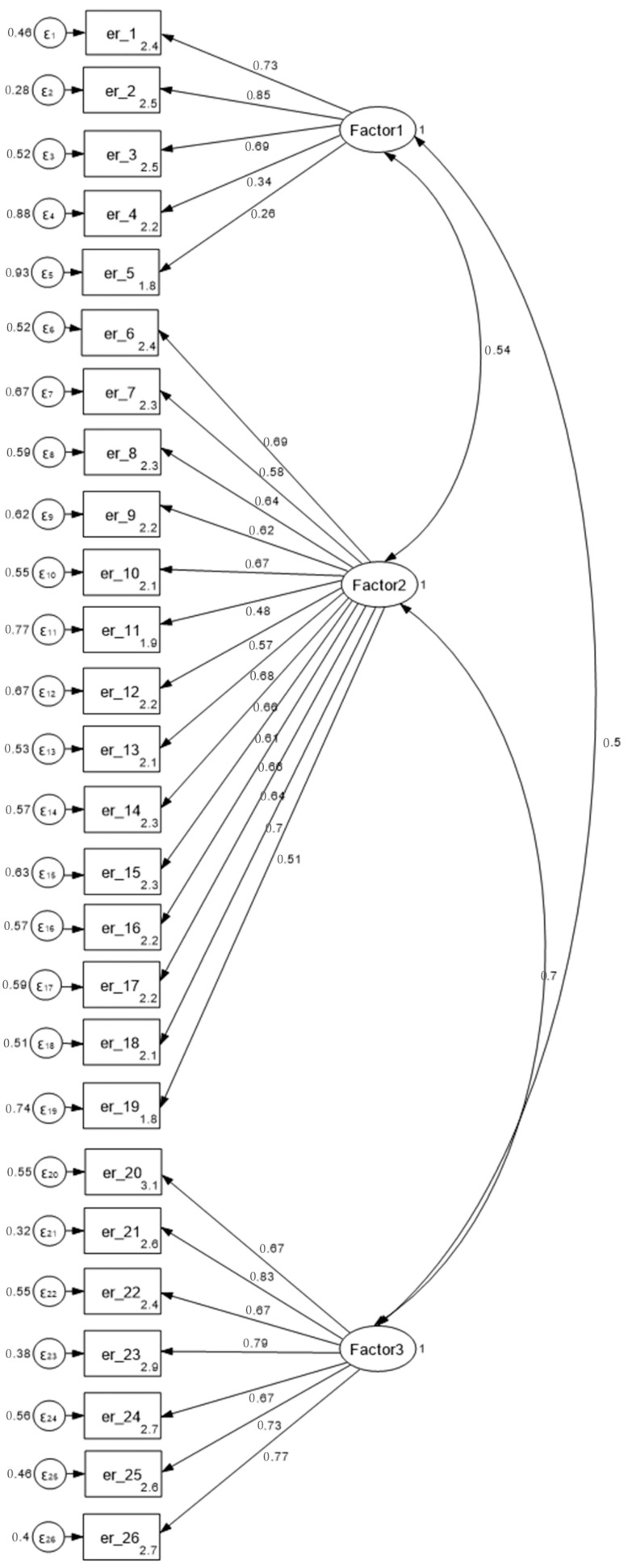
Confirmatory Factor Analysis (CFA) model of the Greek version of the Parental Stressor Scale: Neonatal Intensive Care Unit (PSS:NICU). *Note*: Standardized coefficients are depicted in the figure. Maximum likelihood method was used.

To evaluate the three-factor structure of the PSS:NICU, a confirmatory factor analysis (CFA) was performed. Sampling adequacy and suitability for factor analysis were first assessed using the KMO measure and Bartlett’s test of sphericity. The KMO value was 0.86, and Bartlett’s test was significant (*p* < 0.001), confirming the appropriateness of the data for CFA. The results are presented in Table 3 and illustrated in Figure 1. All standardized factor loadings were significant (*p* < 0.001), ranging from 0.26 to 0.85 for Sights and Sounds, 0.48 to 0.70 for Infant Behavior and Appearance, and 0.67 to 0.83 for Parental Role Alteration. Model fit indices indicated an acceptable fit: χ^2^/df = 1.59, SRMR = 0.077, CFI = 0.92, TLI = 0.90 and RMSEA = 0.063 (90% CI: 0.05–0.07). Overall, these results support the adequacy of the three-factor structure of the questionnaire.

The mean overall stress score was 2.86 (SD = 0.73), as shown in Table 4. Subscale mean scores were 2.54 (SD = 0.75) for Sights and Sounds, 2.73 (SD = 0.83) for Infant Behavior and Appearance, and 3.36 (SD = 0.97) for Parental Role Alteration. Significant positive correlations were observed among all subscales (r = 0.39–0.63, *p* < 0.001) and between each subscale and the total score (r = 0.64–0.94, *p* < 0.001), supporting the internal consistency and coherence of the Greek version of the PSS:NICU.

To further explore potential group differences, comparisons were conducted by parental sex and reason for NICU admission. Differences according to parental sex were examined as part of the assessment of discriminant validity. Male participants reported significantly lower scores than females on the Infant Behavior and Appearance (*p* = 0.001) and Parental Role Alteration (*p* < 0.001) subscales, as well as on the overall stress score (*p* < 0.001). No significant gender difference was observed for the Sights and Sounds subscale (*p* = 0.377) (Table 5). In addition, admission to the NICU due to low birth weight was significantly associated with higher scores on the Sights and Sounds subscale (M = 2.84, SD = 0.54 vs. M = 2.50, SD = 0.77; p = 0.019).

## 4. Discussion

To our knowledge, this is the first study to translate, culturally adapt, and psychometrically validate the PSS:NICU in the Greek context, thereby filling an important gap in neonatal and psychosocial research in Greece. Although the PSS:NICU has previously been translated and used in Greek settings [30,31], no prior study has undertaken a systematic process of cultural adaptation and psychometric validation, limiting comparability with international research. The present study establishes the Greek version of the PSS:NICU as a reliable and valid instrument for assessing parental stress and reinforces the cross-cultural robustness of the scale across diverse healthcare systems. By providing a standardized, psychometrically sound tool, this study supports clinicians and researchers in implementing evidence-based screening and facilitating international research collaborations, thereby advancing family-centered care practices in Greek NICUs.

The CFA supported the original three-factor structure of the PSS:NICU—sights and sounds, infant behavior and appearance, and parental role alteration—with model fit indices within acceptable to good thresholds (χ^2^/df = 1.59; CFI = 0.92; TLI = 0.90; RMSEA = 0.063; SRMR = 0.077). These findings align with those reported by Franck et al. [21] in the UK (CFI = 0.94, RMSEA = 0.06), Montirosso et al. [13] in Italy (CFI = 0.91, TLI = 0.89), and Beheshtipour et al. [32] in Iran (RMSEA = 0.07), further supporting the scale’s factorial stability across cultures. Findings across multiple countries suggest that, despite differences in healthcare systems and parental expectations, the core dimensions of NICU-related stress appear stable [14,18,29].

The internal consistency of the Greek version was excellent, with a total Cronbach’s alpha of 0.93. Subscale reliability was also high: 0.72 for Sights and Sounds, 0.90 for Infant Behavior and Appearance, and 0.88 for Parental Role Alteration. These values are comparable to those observed in the original study by Miles et al. [10], where subscale alphas ranged from 0.70 to 0.89, and in other validated versions, including Arabic (α = 0.82–0.91), Portuguese (α = 0.76–0.95), and Turkish (α = 0.83–0.94) [11,14,18]. The consistency of these coefficients demonstrates that the Greek version retains both conceptual and psychometric integrity, making it appropriate for longitudinal research and intervention studies.

In terms of parental stress levels, the Parental Role Alteration domain had the highest mean score (M = 3.36, SD = 0.97), echoing findings from international literature. Wigert et al. [33], Heidari et al. [2], and Montirosso et al. [13] similarly identified this domain as the most distressing for parents, while Franck et al. [21] showed that British parents also rated role alteration as the most stressful aspect of the NICU experience. This consistent pattern underscores a universal theme: when parents are unable to hold, feed, or comfort their neonate, they experience distress stemming from disrupted caregiving identity. The prominence of this domain in Greece may also reflect cultural norms, where close physical proximity and active caregiving are highly valued components of parental identity, thus amplifying the stress of separation and restricted involvement [34,35].

The mean overall stress score in this study (M = 2.86, SD = 0.73) falls within the moderately high range and is comparable to findings in other contexts, such as 2.79 [21] and 2.91 [32]. This similarity suggests that elevated stress among NICU parents is a consistent global phenomenon, although its intensity may vary depending on sociocultural norms, family expectations, and healthcare system structures. In Greece, where family bonds and caregiving roles are culturally emphasized, the prominence of Parental Role Alteration as the most stressful domain may be particularly relevant.

Convergent validity of the Greek PSS:NICU was supported through significant correlations among all three subscales (r = 0.39–0.63) and between subscales and the total score (r = 0.64–0.94), consistent with the Japanese validation [12]. These intercorrelations confirm that, although each subscale captures a distinct stress dimension, together they reflect a unified construct of the NICU experience, supporting both domain-specific and overall assessment of parental stress.

In line with previous research emphasizing the use of validated psychodiagnostic tools to assess parental distress in NICU settings, the Greek version of the PSS:NICU complements existing measures of stress and psychological adjustment by providing a culturally adapted and psychometrically robust option for clinical and research use. Beyond statistical confirmation, these findings provide important cultural insights into the experiences of parents in Greek NICUs, where stress appears to be particularly associated with alterations in the parental role and limited opportunities for direct caregiving. This pattern underscores the importance of family-centered care practices and highlights the potential benefit of psychosocial interventions that enhance parental participation, emotional support, and communication with healthcare professionals. Furthermore, by offering a culturally adapted and psychometrically sound Greek version of the PSS:NICU, this study extends the applicability of the instrument to both clinical practice and international research. Collectively, these findings enrich existing knowledge by demonstrating that while the core domains of parental stress are universal, their intensity and contextual expression may differ across cultural and healthcare settings.

This study also presents several notable strengths. It followed rigorous, internationally accepted procedures for translation and cross-cultural adaptation, including forward–backward translation, expert review, and pilot testing. Psychometric evaluation was comprehensive, employing both Cronbach’s alpha and McDonald’s omega coefficients to ensure internal consistency, as well as confirmatory factor analysis to verify construct validity. The inclusion of both mothers and fathers from multiple hospitals enhanced the representativeness and generalizability of the findings within the Greek context.

Several limitations should also be acknowledged. First, although the sample size was adequate for validation purposes, it was geographically limited to three hospitals in Greece, which may not fully capture the diversity of parental experiences across different regions. Second, the study included a higher proportion of mothers than fathers, which may have limited the statistical power to fully explore gender differences in stress responses. Third, while convergent validity was supported through intercorrelations among subscales and preliminary evidence of discriminant validity was provided by gender-based differences in scores, external standardized measures of parental stress, anxiety, or depression were not administered, restricting the broader assessment of construct validity. Finally, the cross-sectional design precluded the evaluation of temporal stability and test–retest reliability, which are important indicators of measurement consistency over time. Future studies should aim to recruit larger and more geographically diverse samples, achieve a more balanced representation of mothers and fathers, and employ longitudinal designs incorporating additional validated psychological measures to strengthen construct validation.

### Clinical Implications

The validation of the PSS:NICU in Greek provides clinicians with a standardized and culturally appropriate tool for assessing parental stress during neonatal hospitalization. Routine use of the instrument can help NICU staff identify parents experiencing high levels of stress, particularly in the domain of parental role alteration and enable early referral for psychological support or counseling. By highlighting specific sources of distress—such as separation from the neonate or inability to participate in caregiving—the tool can guide targeted interventions that promote parental involvement, strengthen bonding, and reduce long-term psychological consequences. Furthermore, the availability of a validated Greek version facilitates the incorporation of stress assessment into family-centered care practices, contributing to improved communication between healthcare professionals and parents, and ultimately enhancing both parental well-being and neonatal outcomes. The instrument can also serve as a reliable outcome measure for evaluating the effectiveness of psychosocial and family-support interventions in NICU settings.

## 5. Conclusions

This study provides the first systematic translation, cultural adaptation, and psychometric validation of the PSS:NICU in the Greek context. The Greek version demonstrated excellent internal consistency (α = 0.93) and supported the original three-factor structure—Sights and Sounds, Infant Behavior and Appearance, and Parental Role Alteration—through CFA. Among these, Parental Role Alteration was identified as the most stressful domain (M = 3.36, SD = 0.97), consistent with international findings, reflecting the strong emotional impact of limited parental involvement in neonatal care. These results underscore the universal relevance of maintaining parental identity and participation during NICU hospitalization. Beyond its psychometric contribution, the validated Greek version of the PSS:NICU offers an evidence-based tool for routine clinical screening, enabling healthcare teams to identify parents at higher risk of psychological distress and tailor psychosocial interventions accordingly. By integrating this measure into clinical practice, NICUs can strengthen family-centered care, improve communication, and enhance parental well-being as part of holistic neonatal care.

## Figures and Tables

**Table 1:** Sample characteristics (N = 150).

		N (%)
Parent	Mother	89 (59.3)
	Father	61 (40.7)
Age (years)	18–30	41 (27.3)
	31–40	83 (55.3)
	41–50	26 (17.3)
Family status	Unmarried	2 (1.3)
	Married	137 (91.3)
	Civil partnership	11 (7.3)
Educational status	Primary/Middle school	3 (2.0)
	High school	26 (17.3)
	University	47 (31.3)
	Technical Educational Institute	42 (28.0)
	MSc	25 (16.7)
	PhD	3 (2.0)
	Other	4 (2.7)
Nationality	Greek	149 (99.3)
	Other	1 (0.7)
Employment	Unemployed	12 (8.0)
	Employee in private sector	76 (50.7)
	Employee in public sector	36 (24)
	Freelancer	22 (14.7)
	Other	4 (2.7)
Having another child		41 (27.3)
If yes, how many	1	31 (77.5)
	2	5 (12.5)
	3	4 (10)
Prior experience to NICU		8 (5.3)
Mode of delivery	Vaginal	13 (8.7)
	C-section	137 (91.3)
Amniotic fluid	Clear	142 (95.9)
	Colored	6 (4.1)
Neonate gender	Male	44 (29.3)
	Female	106 (70.7)
Reason for NICU admission	Preterm delivery	124 (82.7)
	Low birth weight	20 (13.3)
	Respiratory problems	22 (14.7)
	Other reason	15 (10.0)
		Mean (SD)
Gestational age (weeks)		32.1 (3.3)
Birth weight (grams)		1761.2 (641.9)
Body length (cm)		43 (4.9)
Head circumference (cm)		40.7 (24.7)
Max O_2_		44.7 (16.5)
		Median (IQR)
APGAR 1 min		8 (7–9)
APGAR 5 min		10 (9–10)

cm = centimeters; IQR = interquartile range; min: minutes; NICU = neonatal intensive care unit; SD = standard deviation.

**Table 2:** Descriptive statistics of PSS:NICU items: mean (standard deviation), corrected item–total correlations and reliability coefficients (Cronbach’s α if item deleted).

Factor	Item	Mean ^1^ (SD)	Corrected Item-Total Correlation	Cronbach’s Alpha If Item Deleted
Sights and sounds	1	2.49 (1.03)	0.66	0.59
	2	2.71 (1.07)	0.60	0.61
	3	3.05 (1.19)	0.42	0.69
	4	2.48 (1.11)	0.39	0.70
	5	2.01 (1.12)	0.33	0.72
Infant behavior and appearance	6	3.01 (1.25)	0.64	0.89
	7	2.93 (1.29)	0.55	0.89
	8	2.61 (1.16)	0.61	0.89
	9	2.86 (1.30)	0.56	0.89
	10	2.58 (1.25)	0.66	0.89
	11	2.11 (1.10)	0.47	0.90
	12	2.74 (1.22)	0.55	0.89
	13	2.81 (1.35)	0.64	0.89
	14	2.65 (1.17)	0.61	0.89
	15	3.13 (1.38)	0.57	0.89
	16	3.01 (1.36)	0.61	0.89
	17	2.85 (1.32)	0.60	0.89
	18	2.67 (1.27)	0.66	0.89
	19	2.23 (1.28)	0.50	0.90
Parental role alteration	20	3.64 (1.16)	0.59	0.87
	21	3.26 (1.27)	0.76	0.85
	22	3.07 (1.31)	0.59	0.88
	23	3.45 (1.21)	0.74	0.86
	24	3.43 (1.30)	0.61	0.87
	25	3.34 (1.25)	0.68	0.86
	26	3.35 (1.22)	0.72	0.86

Note: Items rated on a 5-point Likert scale from 1 = Not at all stressful to 5 = Extremely stressful. N/A responses were treated according to metric 2 of the scoring system. ^1^ Mean scores represent average stress level per item; higher scores indicate greater stress.

**Table 3:** Indices from CFA.

Fit index Type	Observed Value	Acceptable Value
Chi-square/df	1.59	<3
Standardized Root Mean Squared Error (SRMR)	0.077	≤0.08
Comparative Fit Index (CFI)	0.92	≥0.90
Tucker–Lewis Index (TLI)	0.90	≥0.90
RMSEA (root mean square error of approximation)	0.063	≤0.08

**Table 4:** Descriptive statistics, intercorrelations, and reliability indices of the Greek version of the PSS:NICU.

				Pearson’s Correlation Coefficients				Cronbach’s Alpha	Omega Coefficient
		Mean	SD	1.	2.	3.	4.		
1.	Sights and sounds	2.54	0.75	1.00	0.48 ***	0.39 ***	0.64 ***	0.72	0.72
2.	Infant behavior and appearance	2.73	0.83		1.00	0.63 ***	0.94 ***	0.90	0.90
3.	Parental role alteration	3.36	0.97			1.00	0.83 ***	0.88	0.88
4.	Summary stress score	2.86	0.73				1.00	0.93	0.93

*** *p* < 0.001.

**Table 5:** Participants’ PSS:NICU scores by gender.

	Gender				*p* Student’s *t*-Test
	Women (n = 89)		Men (n = 61)		
	Mean	SD	Mean	SD	
Sights and sounds	2.59	0.74	2.48	0.77	0.377
Infant behavior and appearance	2.90	0.81	2.47	0.80	0.001
Parental role alteration	3.63	0.92	2.96	0.89	<0.001
Summary stress score	3.04	0.68	2.60	0.72	<0.001

## Data Availability

The data presented in this study are available on reasonable request from the corresponding author. The data are not publicly available due to ethical restrictions related to participant confidentiality.
